# Supplementary material for: Causal Relationship between Aging and Anorexia Nervosa: A White-Matter-Microstructure-Mediated Mendelian Randomization Analysis
Source: Biomedicines. 2024 Aug 16;12(8):1874. doi: 10.3390/biomedicines12081874 (PMC11351342; doi:10.3390/biomedicines12081874)
Supplement: Supplementary file 1 [file biomedicines-12-01874-s001.zip › Supplementary Figure/Figure S8/Leaveoneout plot of MR analysis for TL to ubm-b-1967.pdf]

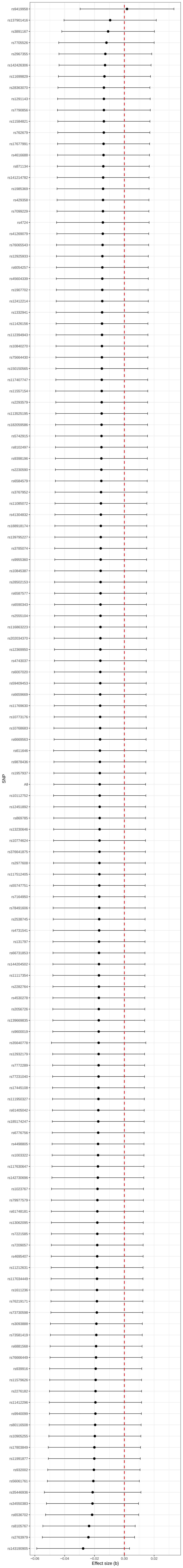
rs9419958
rs137901416
rs3891167
rs7705526
rs2967355
rs142426306
rs11699829
rs28363070
rs1291143
rs7790856
rs11584821
rs762679
rs17677991
rs4616688
rs871134
rs141214782
rs1985369
rs429358
rs7099229
rs4724
rs41269079
rs76065543
rs12925933
rs6054257
rs45604339
rs1907702
rs12412214
rs1332941
rs11426156
rs112394943
rs10840270
rs75664430
rs150150565
rs117407747
rs11557154
rs2293579
rs113525195
rs182059586
rs5742915
rs8102497
rs9398196
rs2230590
rs6584579
rs3767952
rs11085072
rs41304832
rs188918174
rs139795227
rs3785074
rs9955360
rs10845387
rs28502153
rs6587577
rs6590343
rs2555104
rs116863223
rs202034370
rs12369950
rs4743037
rs6007020
rs59409453
rs6659669
rs11769630
rs10773176
rs10768683
rs6669563
rs611646
rs9878436
rs1957937
All
rs10112752
rs12451892
rs869785
rs13230646
rs10774624
rs376641875
rs2977608
rs117512405
rs55747751
rs7164950
rs78491606
rs2538745
rs4731541
rs131797
rs66731853
rs144204502
rs11117354
rs2282764
rs4530278
rs2056726
rs139669835
rs9600019
rs35640778
rs12932179
rs7772289
rs77231040
rs17445108
rs111950327
rs61405042
rs185174247
rs6776756
rs4498805
rs1003322
rs117630647
rs142730696
rs1023767
rs79977579
rs61748181
rs13062095
rs7221585
rs7209057
rs4695407
rs11212631
rs117034449
rs1611236
rs76219171
rs73730598
rs3093888
rs73581419
rs6881568
rs76666449
rs939916
rs11579626
rs2276182
rs11412296
rs9940099
rs80116508
rs10905255
rs17803849
rs11991877
rs932002
rs56061761
rs35446936
rs34550383
rs6536702
rs8105767
rs2763979
rs143190905
SNP
−0.06
−0.04
−0.02
0.00
0.02
Effect size (b)
